# Precise scheduling of B-box transcription factors: the core network for balancing plant growth, metabolism and stress resistance

**DOI:** 10.3389/fpls.2026.1809754

**Published:** 2026-04-15

**Authors:** Zhi Luo, Liqin Tu, Yujia Luo, Yunjie Wang, Lihu Wang, Fenfen Yan

**Affiliations:** 1 College of Horticulture and Forestry, Tarim University, Alar, China; 2 Handan Academy of Agricultural Sciences, Handan, Hebei, China; 3 School of Landscape and Ecological Engineering, Hebei University of Engineering, Handan, Hebei, China

**Keywords:** BBX proteins, growth and development, photomorphogenesis, secondary metabolite, stress

## Abstract

The B-box (BBX) zinc finger transcription factor family functions as a central regulatory hub in plants, responsible for perceiving and integrating environmental signals to coordinate growth, development, and stress adaptation. Typically, members of this family typically contain one or two conserved B-box domains, with certain members also containing CCT domains or VP motifs, these multi-domain architectures underlie the functional diversity of BBX proteins. This review summarizes the key roles of BBX proteins across the plant life cycle—including growth regulation, secondary metabolism, photomorphogenesis, and responses to biotic and abiotic stresses. Given the distinct regulatory capacity of BBX proteins in harmonizing environmental signals with crucial agronomic traits in horticultural crops, such as stress resilience and secondary metabolite production, further investigation is needed to decipher functional redundancy and interaction networks among BBX members, elucidate their integrated response mechanisms under complex field conditions. Analyzing these crucial regulatory networks will establish a fundamental framework and molecular targets for precisely manipulating the core domains or interaction interfaces of BBX proteins, thereby improving crop quality and resilience through approaches like gene editing, thus promoting their effectiveness in precision breeding.

## 1 Introduction

As fixed-growing organisms, higher plants remain in a single location throughout their entire life cycle and are unable to actively avoid adverse environments by moving. To maintain survival and reproduction, plants have evolved a sophisticated and complex regulatory network, thereby achieving a dynamic balance between growth and development and environmental adaptation (Wu et al., 2025). Among the numerous families of transcription factors, B-box proteins (BBX) are widely involved in processes such as plant perception of light signals, biological clock regulation, and stress response. They interact with other regulatory factors (such as HY5, COP1, PIFs, etc.) to form a complex regulatory network, precisely guiding seeds from breaking through the soil to flowering and fruiting, from pigment accumulation to initiating defense mechanisms to resist invasion (Song et al., 2020; Luo et al., 2023; Gao et al., 2024; Li et al., 2026; Li et al., 2025; Xie et al., 2025). The functions of BBX proteins run through multiple key stages of the plant life cycle, profoundly influencing the plant’s morphology, physiological state, and environmental adaptability. Therefore, a thorough understanding of the regulatory mechanism of BBX proteins not only enriches our knowledge of plant environmental adaptation strategies but also provides valuable theoretical basis and genetic resources for horticultural crop molecular genetic improvement. By precisely manipulating the expression or function of specific BBX genes, it is expected to collaboratively optimize the growth and development and resistance (such as plant architecture, flowering period, and fruit color) and stress resistance (salt, alkali, drought, cold resistance, and disease and pest resistance) of horticultural crops, thereby cultivating new varieties that are high-yielding, high-quality, and stable-yielding.

Although, significant progress has been achieved in the study of the B-box (BBX) transcription factor family. However, existing reviews predominantly concentrate on a singular aspect. Some reviews focus on photomorphogenesis and flowering regulation mechanisms in the model plant *Arabidopsis thaliana* (Gangappa and Botto, 2014; Song et al., 2020; Song et al., 2024; Lira et al., 2026), while others emphasize the role of BBX proteins in abiotic stress responses or analyze their structural diversity from an evolutionary standpoint (Crocco and Botto, 2013; Jiang et al., 2025). These studies often compartmentalize the various functions of BBX proteins—such as growth and development, secondary metabolism, light signal transduction, and stress response—resulting in a lack of a systematic understanding of their role as an “integration hub.” Notably, current reviews have inadequately addressed the unique regulatory functions of BBX proteins in horticultural crops, including trees and vegetables. Furthermore, they have not thoroughly investigated the comprehensive response mechanisms of BBX proteins in fruit quality formation, including pigment accumulation and specialized metabolism, as well as their responses to complex adverse stresses in controlled cultivation environments.

This review aims to integrate the multifunctional roles of BBX proteins throughout the plant life cycle, systematically elucidating their key regulatory functions and molecular mechanisms in growth and development, including seed germination, plant architecture, and flowering regulation. It also addresses the accumulation of secondary metabolites, such as anthocyanins, carotenoids, and pharmaceutical compounds, as well as responses to biotic and abiotic stresses. In particular, the review highlights recent advancements in the regulation of significant agronomic traits in horticultural crops and explores the central role of BBX proteins as a node for signal integration between light and hormone signaling pathways. Future research directions and application prospects in this domain are also discussed. It is suggested that subsequent studies should concentrate on analyzing the functional network of BBX proteins in complex environments, clarifying the molecular basis of their pleiotropic effects, and investigating methods to precisely target key domains or interaction sites of BBX proteins through approaches such as gene editing. This will provide a theoretical foundation and practical targets for crop improvement and precision breeding, thereby establishing a robust theoretical basis for scientific research and breeding practices in related fields.

## 2 Origin and structure of BBX

**Figure 1:**
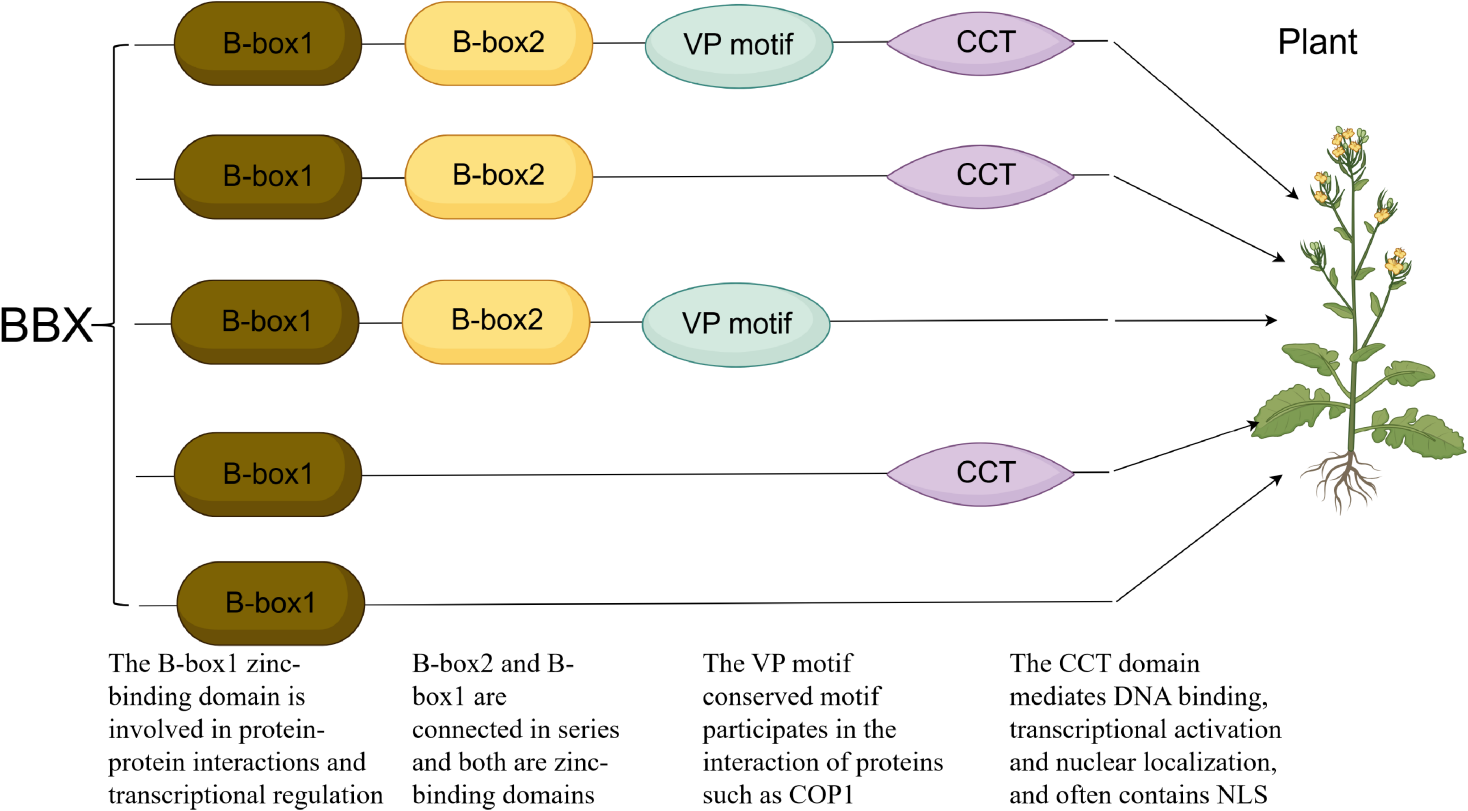
Structural characteristics of the BBX gene.

B-box proteins (BBX) are a class of zinc-binding transcription factors or regulatory factors containing B-box domains, which play a key role in plant light signal transduction and various developmental processes. According to the composition of their domains, plant BBX proteins can be classified into five structural groups. Its typical feature is that the N-terminal contains one or two concatenated B-box domains. Some members also have conserved CCT (CO, Co-like, TOC1) domains and/or valine-proline (VP) motifs at the C-terminal (**Figure 1**). The B-box domain is approximately 40 amino acid residues long and can bind to two zinc ions. Its conserved cysteine (Cys), histidine (His), and aspartic acid (Asp) residues are crucial for maintaining structural stability (Massiah et al., 2006; Crocco and Botto, 2013). This domain is directly involved in protein-protein, protein-DNA interactions and transcriptional regulation (Datta et al., 2007; Datta et al., 2008; Qi et al., 2012; Gangappa et al., 2013a; Gangappa et al., 2013b). In addition, based on the common sequence and the spacing of zinc coordination residues, B-boxes can be classified into two types: B-box1 (B1) and B-Box 2 (B2). In plants such as Arabidopsis thaliana, proteins containing double B-boxes are usually arranged in tandem with B1 and B2 (Crocco and Botto, 2013). The CCT domain is composed of approximately 43 amino acids and is highly conserved. Its main functions include mediating DNA binding, transcriptional activation, nuclear localization, and protein interactions (Robson et al., 2001; Jang et al., 2008; Gendron et al., 2012; Gangappa et al., 2013b). For instance, CO/BBX1 directly binds to the FT promoter through its CCT domain to regulate flowering (Yan et al., 2011). In addition, nuclear localization signals (NLSs) often serve as part of the CCT domain, guiding BBX proteins into the nucleus (Robson et al., 2001; Tiwari et al., 2010; Gendron et al., 2012; Gangappa et al., 2013b). The VP motif (conserved sequence is G-I/VV-P-S/T-F) exists at the C-terminal of some BBX proteins, is close to the CCT domain, and participates in the interaction with proteins such as COP1 (Holm et al., 2001; Datta et al., 2006; Tiwari et al., 2010). The multiple structures of B-box endow it with functional diversity, safeguarding the life cycle of plants.

From an evolutionary perspective, the BBX protein family originated in the early days of green plants. Phylogenetic analysis of 214 BBX proteins from 12 plant species (green algae, mosses, lycopodonts, monocotyledons and dicotyledons) indicates that the five structural groups independently evolved in the early stage of green plant evolution. The B-box core sequences of all structure groups retain a common and conserved domain topology. The study also identified seven novel motifs specific to each structural group (Crocco and Botto, 2013). During the process of evolution, the amino acid sequences of the two B-box domains are strongly constrained and conserved, but the NLSs and VP motifs at the C-terminal show radiative variations, which may be an important basis for the functional diversity of BBX proteins in green plants (Crocco and Botto, 2013; Kim et al., 2013). In addition, genomic comparative analysis suggests that the B1 and B2 motifs are likely to have originated from ancient segment duplication and internal deletion events (Crocco and Botto, 2013). Double B-box proteins have been found in single-celled green algae, indicating that the replication event of B-box occurred at least 450 million years ago (late Silurian), much earlier than the plant landing (Kenrick and Crane, 1997; Crocco and Botto, 2013). BBX proteins have long existed and rapidly expanded in the plant kingdom, indicating that they play a key role in the process of plants adapting to the environment.

## 3 BBX involvement in plant growth and development

**Figure 2:**
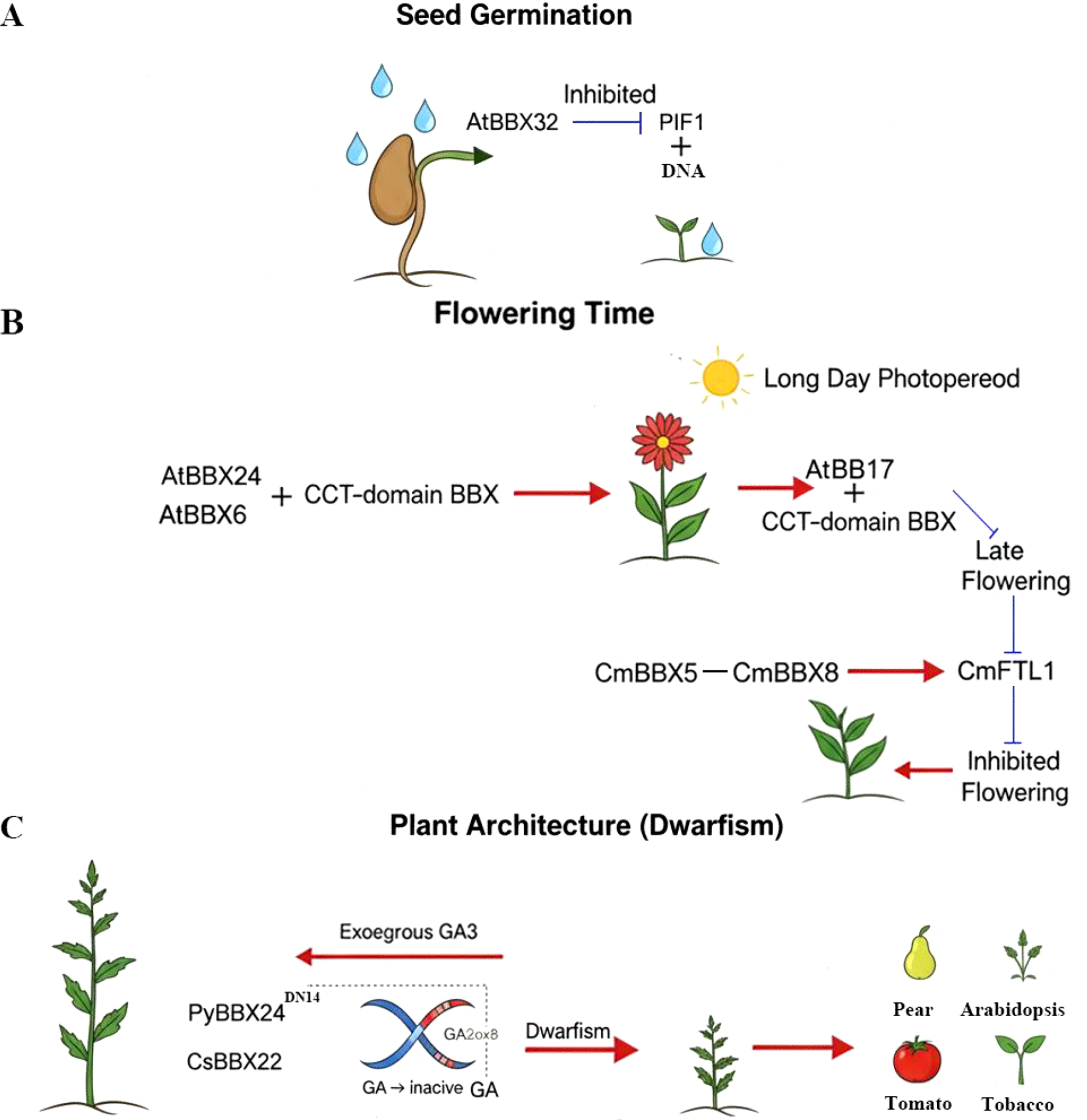
BBX promotes plant growth and development. **(A)** BBX protein participates in seed germination **(B)** BBX protein participates in plant flowering **(C)** BBX protein participates in plant plant type. The plus sign in the figure indicates binding, the t-line indicates inhibition, and the red arrow indicates promotion.

The functions of BBX transcription factors span multiple key stages from seed germination to reproductive development, forming a complex regulatory network. During the seed germination stage, the BBX protein is a key integrator of environmental signals. For instance, Arabidopsis BBX32 (containing only the B-box1 structure) was identified as a positive regulatory factor for seed germination stimulated by water absorption (**Figure 2A**),enabling plants to coordinate water and timely initiate the germination process (Gao et al., 2024). Not only that, in terms of regulating the flowering time, the functions of BBXs are particularly prominent and diverse (**Figure 2B**). The BBX protein in the CCT domain is the core integrator of the photoperiodic flowering pathway, directly activating the expression of the florin gene FT under long-day conditions and promoting flowering (Yang et al., 2025). In the BBX family, different members can precisely regulate the activity of CCT or the transcription of FT genes, but there is a significant functional differentiation in their regulatory patterns. One type of members act as transcriptional activators, such as AtBBX6 and AtBBX24 (Hassidim et al., 2009; Li et al., 2014). Another category acts as inhibitory factors, including AtBBX32 (Takase et al., 2011; Tripathi et al., 2017), BBX17 and CmBBX5. For instance, in Arabidopsis thaliana, BBX17 can bind to CCT and inhibit its function, thereby causing delayed flowering (Xu et al., 2022b); In chrysanthemums, CmBBX5 interacts with CmBBX8 to interfere with the activation ability of the target gene *CmFTL1*, thereby inhibiting the flowering process (Wang et al., 2024). This interaction network ensures the precise adaptation of the flowering time to the external environment. In addition to regulating germination and flowering, BBX proteins (B-box domain proteins) have been identified as key transcription factors controlling plant dwarfing traits. They can reduce the levels of biologically active GA in plants by activating the expression of GA degradation enzyme genes (e.g., GA2ox), thereby inducing dwarfing phenomena (**Figure 2C**). In citrus, BBX22 has been shown to directly bind to and activate the promoter of the *GA2ox8* gene, leading to GA inactivation. Overexpression of *CsBBX22* can significantly shorten the internode length of citrus, sweet orange and tomato, while exogenous application of GA3 can completely reverse this dwarfization phenotype, directly demonstrating its effect through the GA pathway (Fu et al., 2024). In pears, a function-acquired mutant PyBBX24^DN14^(mutation results in an incomplete VP motif) caused by 14-bp deletion has a truncated protein that can more effectively directly bind to and activate *PyGA2ox8*, thereby inducing stable dwarfism phenotypes in multiple species such as pears, *Arabidopsis thaliana*, tobacco, and tomatoes (Yang et al., 2024). The mechanism by which BBX-GA2OX module can cause dwarfism in multiple plants is that BBX can directly bind to the GA2ox8 promoter to activate its expression, thereby inactivating the bioactive gibberellin (GAs) and reducing plant height. This highlights the conservation and powerful efficacy of the BBX-GA2ox module in cross-species dwarfism regulation. In conclusion, the BBX gene family plays a significant role in seed germination, flowering time and plant type composition.

## 4 BBXs are involved in plant secondary metabolites

Plant B-box (BBX) zinc finger proteins, as key transcriptional regulatory hubs, have far exceeded their fundamental regulatory roles in photomorphogenesis and flowering timing. They have demonstrated powerful and diverse functions in regulating the biosynthesis of important secondary metabolites such as anthocyanins and carotenoids, profoundly influencing the coloring, quality and nutritional value of fruits.

### 4.1 Fruit colorist-the vibrant world created by BBXs regulation of anthocyanins

**Figure 3:**
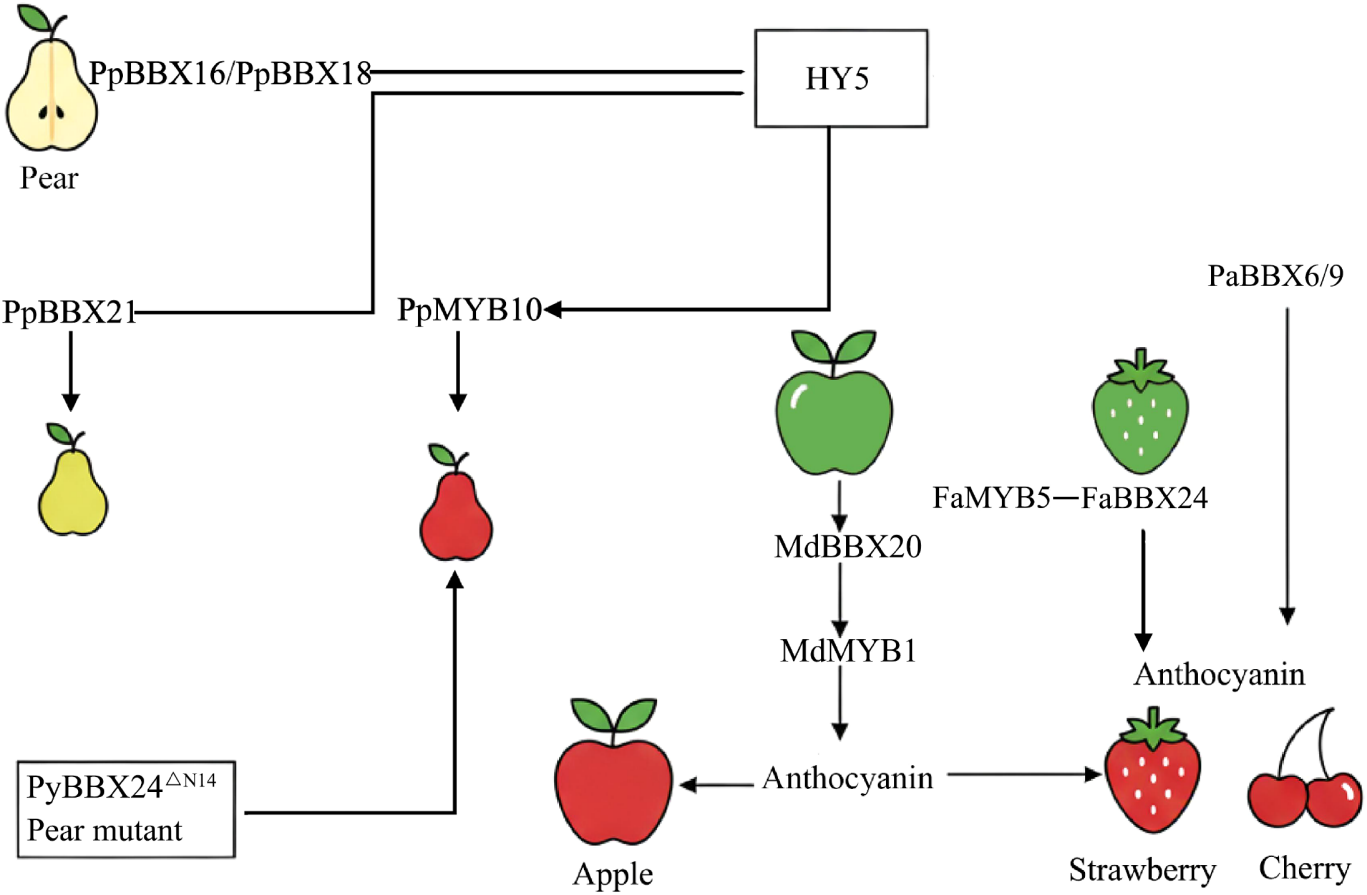
BBX involvement in the anthocyanin biosynthesis pathway in fruit. The arrows in the figure represent promoting effects, the line segment indicates an interaction between two proteins.

The BBX protein is the core component of the regulatory network for anthocyanin biosynthesis in plants, and its function is mainly achieved through the core regulatory module of BBX-HY5-MYB. Existing studies have shown that members of this family play a key role in various fruit trees. For instance, in pears PpBBX16 and PpBBX18 can interact with the core factor of light signal PpHY5, thereby activating the expression of the key regulatory gene PpMYB10 and positively promoting the accumulation of anthocyanins. However, interestingly, PpBBX21 inhibits this pathway by competitively binding to PpHY5, forming a precise antagonistic regulation that precisely controls fruit coloring (Bai et al., 2019a; Bai et al., 2019b). In apples, MdBBX20 has been found to promote anthocyanin synthesis in the peel by activating downstream genes such as MdMYB1 (Fang et al., 2019). In addition, FaBBX24 in strawberries and PavBBX6/9 in sweet cherries have all been confirmed to be positive regulatory factors for anthocyanin synthesis (Wang et al., 2023; Zhang et al., 2023). In addition, naturally occurring interesting mutants provide materials for studying the structure and function of the BBX gene. For instance, PyBBX24^△N14^ in pears (mutation results in an incomplete VP motif) has acquired a strong anthocyanin activation ability due to the structural change at the C-terminal of its protein. This reveals the significance of the relationship between the structure and function of the BBX protein and its application potential in breeding (Yang et al., 2024). Different BBX members precisely regulate the light response genes through collaborative activation and competitive antagonism, achieving flexible regulation of the developmental plasticity of anthocyanin accumulation (**Figure 3**).

### 4.2 Pleiotropic effects of BBXs: from fruit coloration to the enrichment of medicinal compounds

**Figure 4:**
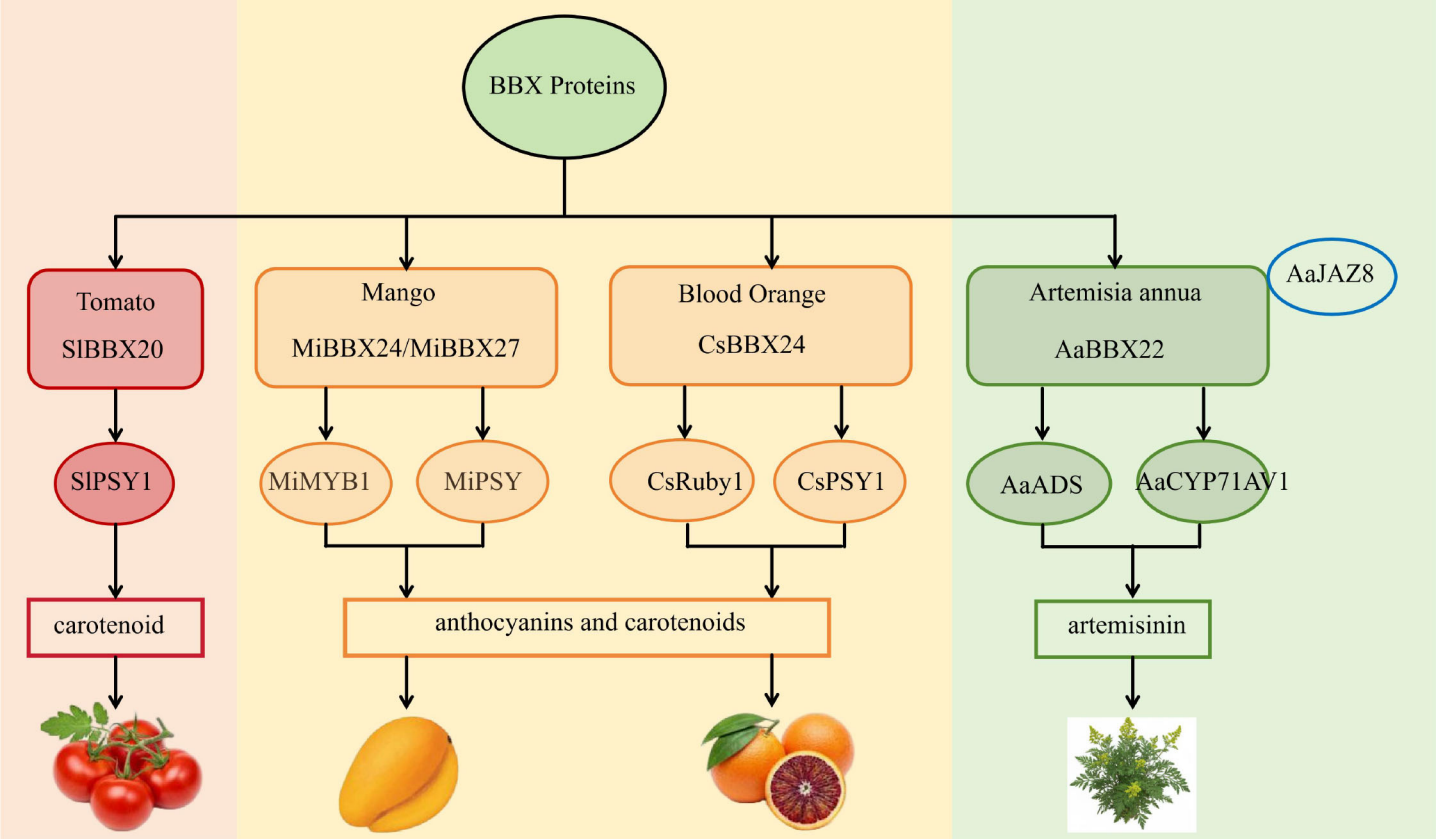
BBX-mediated secondary metabolism (carotenoid and medicinal material synthesis). The arrows in the figure represent promoting effects.

In addition to its crucial role in anthocyanin synthesis, the regulatory network of BBX proteins has also expanded into the field of carotenoid biosynthesis. For instance, tomatoes SlBBX20 has been classically proven to directly bind to the promoter of the key carotenoid synthesis gene SlPSY1, directly driving its expression and promoting the accumulation of carotenoids in fruits (Xiong et al., 2019). More strikingly, recent studies have discovered multiple dual-functional BBX proteins that can synergistically regulate anthocyanins and carotenoids. For instance, MiBBX24 and MiBBX27 in mangoes can simultaneously activate the anthocyanin regulatory factor MiMYB1 and the key carotenoid enzyme gene MiPSY, promoting the synthesis of anthocyanins in the peel and carotenoids in the fruit flesh at the same time (Pan et al., 2025). Similarly, in blood oranges, CsBBX24 can directly bind to and activate the promoters of the carotenoid synthesis gene CsPSY1 and the anthocyanin major gene CsRuby1, coordinating the co-accumulation of the two pigments during fruit ripening. and citrus BBX22 not only influences plant height but also positively regulates carotenoid accumulation in tomato fruits, promoting fruit coloring (Fu et al., 2024; Fu et al., 2025). In addition to participating in the above-mentioned pigment metabolism, the regulatory network of BBX protein further extends to the synthesis of sesquiterpene lides such as artemisinin, which have significant medicinal value. Members such as AaBBX22 identified in Artemisia annua are not only strongly induced and located in the nucleus by jasmonic acid (MeJA) and abscisic acid (ABA), but also can interact with the jasmonic acid signaling pathway inhibitor AaJAZ8. Functionally, AaBBX22 can significantly activate the promoters of key genes in the artemisinin synthesis pathway (such as AaADS and AaCYP71AV1), and positively regulate artemisinin biosynthesis in transgenic plants (He et al., 2023). In summary, the BBX transcription factor family has demonstrated its powerful capabilities and diversity as a universal regulatory hub for plant secondary metabolism, evolving from the precise switch for anthocyanin synthesis to the cooperative dispatcher for carotenoids, and then to the activator for the synthesis of medicinal compounds such as artemisinin (**Figure 4**). Therefore, the function of BBX protein has evolved from the precise regulation of a single pigment pathway to the global regulation of the synergistic accumulation of two pigments. Meanwhile, they play a key role in the regulation of plant secondary metabolism (medicinal compounds).

## 5 BBXs involvement in photomorphogenesis

Plant B-box zinc finger proteins are the core hubs of the optical signal transduction network. By responding to optical signals of different wavelengths and intensities and coordinating the expression of downstream genes, they precisely regulate photomorphogenesis.

The B-box zinc finger protein (BBX) family is the core hub of the plant light signal transduction network. Its members form a complex front-end regulatory network by sensing light signals of specific wavelengths (such as UV-B and red light) and directly interacting with photoreceptors and core signal components. For instance, *Arabidopsis thaliana* BBX11 is upregulated under UV-B and forms a feedback loop with the transcription factor HY5, jointly regulating the synthesis of photoprotective substances to enhance tolerance (Job et al., 2022). In the red light signaling pathway, BBX4 co-locates with photosensitive pigment B (phyB) and photosensitive pigment interaction factor (PIFs) in the nucleus. Phyb-mediated PIFs degradation and BBX4 accumulation jointly inhibit the transcriptional activity of PIF3, thereby promoting (Datta et al., 2006; Heng et al., 2019a). Interestingly, BBX9 serves dual functions. It interacts with phyB and PIFs, phyB stabilizes BBX9, while PIFs inhibit BBX9 transcription, thereby creating a negative feedback loop. Additionally, BBX9 interacts with the positive regulator BBX21, and they mutually activate each other’s transcription, forming a positive feedback loop (Xu et al., 2016). This dynamic interaction with photoreceptors (phyB), key transcription factors (PIFs, HY5), and the positive and negative feedback network it constructs make the BBX protein a key integrator and scheduler of light signals, ensuring the rapid adaptation of plants to fluctuating light environments.

**Figure 5:**
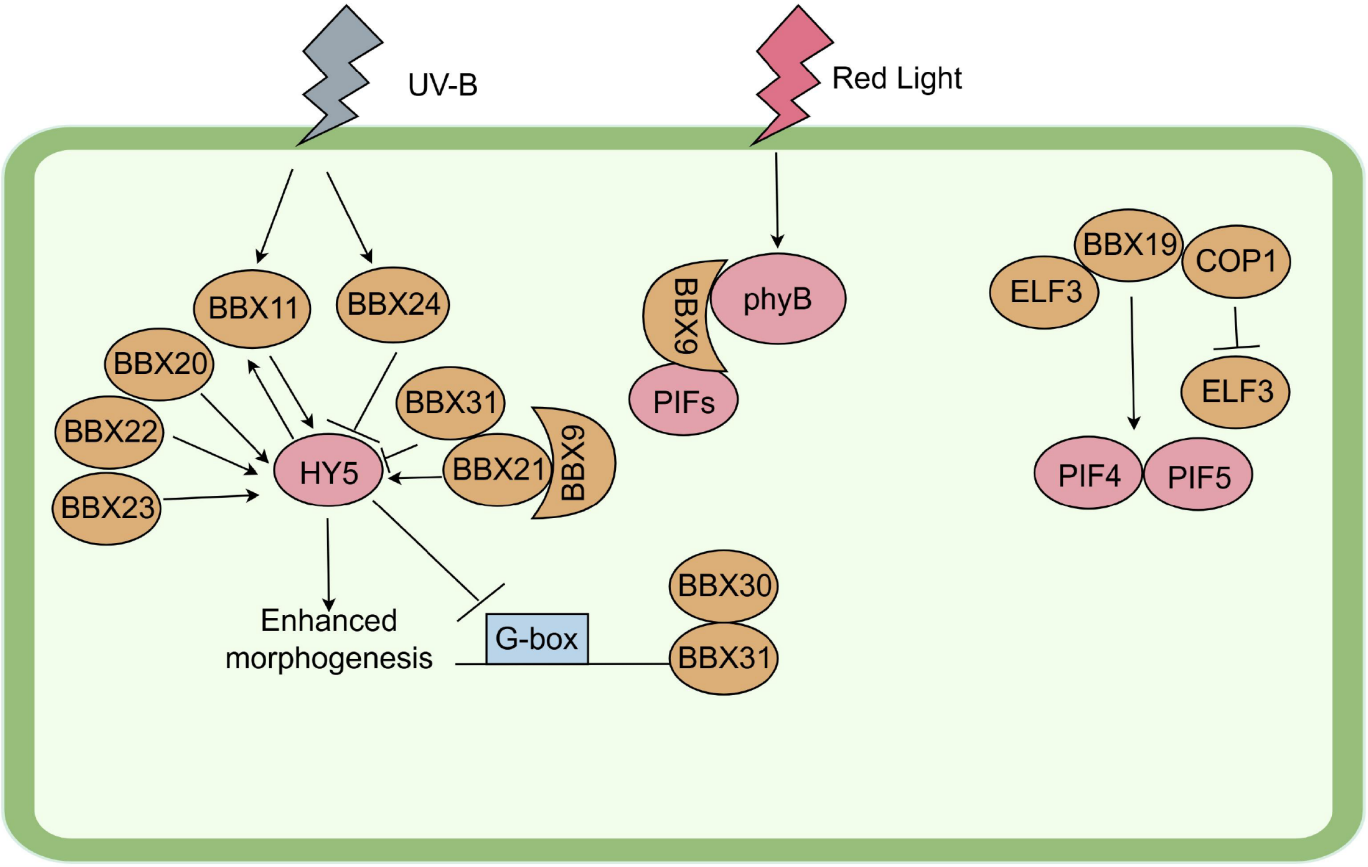
BBX involvement in photomorphogenesis. The arrows in the figure represent promoting effects, T-shaped lines represent inhibitory or degrading effects, and close proximity indicates interaction.

Members of the BBX family exhibit functional differentiation in photomorphogenesis and achieve precise output through multi-level transcriptional regulation and protein stability adjustment. On the one hand, BBX20, BBX21, BBX22, BBX23 and others play a positive regulatory role. BBX21, BBX22 and gibberellin metabolic gene GA2ox1 to activate HY5 expression, and synergize with HY5 to enhance photophogenesis (Datta et al., 2008; Xu et al., 2016; Xu et al., 2017). The effect of BBX22 is particularly prominent under strong light and can interact with HY5 to enhance its activity (Zhang et al., 2017). BBX23 forms a functional heterodimer with HY5 and collaboratively activates downstream genes (Chang et al., 2008). On the other hand, members such as BBX19, BBX24, BBX25, BBX28, BBX30, BBX31 and BBX32 play a negative regulatory role. BBX19 mediates ELF3 degradation by forming complexes with ELF3 and COP1, thereby promoting the expression of PIF4/PIF5 (Wang et al., 2015). BBX24, BBX25 and BBX28 directly bind and inhibit the transcriptional activity of HY5 (Gangappa et al., 2013a; Lin et al., 2018). BBX30 and BBX31, as downstream targets of HY5, their expressions are directly inhibited by HY5 through G-box elements (Heng et al., 2019b; Yadav et al., 2019). BBX32 exerts its effect by binding to and inhibiting the function of the BBX21-HY5 complex (Holtan et al., 2011). The activity of this family is precisely regulated by light-dependent protein stability: in the dark, the COP1-SPA1 complex promotes the degradation of some BBX proteins. In the early stage of light exposure, COP1 is inactivated, and proteins such as BBX21, BBX22, and BBX24 accumulate rapidly (reaching the peak within 3–6 hours). Subsequently, under continuous light exposure, they are gradually degraded through pathways such as COP1 (Chang et al., 2011; Yan et al., 2011; Xu et al., 2016,). In summary, BBX proteins, through their functional diversity, complex interaction networks, and the stability of light-regulated proteins, jointly form a dynamic regulatory system that ensures the precise implementation of photomorphogenesis (**Figure 5**).

## 6 The BBX is involved in plant biotic and abiotic stress

The B-box (BBX) transcription factor, as an important member of the plant zinc finger protein family, has become a key node for regulating plant growth and development as well as environmental adaptation, thanks to its conserved B-box domain and the unique CCT domain of some members. Early research mainly focused on its role in light signal transduction and flowering time regulation (such as AtBBX1 in *Arabidopsis thaliana* and Hd1 in rice). However, an increasing amount of evidence indicates that the BBX family, especially its double B-box (DBB) subfamily without the CCT domain, is widely and deeply involved in the response networks of plants to various biological and abiotic stresses, demonstrating the diversity of gene functions within this family.

### 6.1 The BBX response hub in abiotic stress

Abiotic stress is the main environmental factor that limits crop growth and yield. Research has found that multiple BBX members are specifically induced or inhibited under saline-alkali, drought, low-temperature and high-temperature stresses, and play an important role in plant stress resistance by regulating the expression of downstream stress-related genes. These responses are species and gene-specific, forming a complex regulatory network. Salt and drought stress often trigger osmotic stress and oxidative damage. The BBX gene enhances tolerance by regulating the synthesis of osmotic regulatory substances, ion homeostasis and the antioxidant system (**Table 1**). For instance, although wheat TaDBB4.1 and TaDBB4.2 originated from fragment replication, they functionally differentiated under saline-alkali stress. TaDBB4.1 negatively regulates photosynthetic genes, while TaDBB4.2 positively regulates ion transport and REDOX processes, jointly responding to stress (Du et al., 2026). Tomato SlBBX20 is strongly induced by salt stress, overexpression can significantly improve salt tolerance by maintaining photosynthetic capacity, enhancing the activities of antioxidant enzymes (POD, SOD, CAT), and promoting the expression of the key gene *SlP5CR* for proline synthesis (Ma et al., 2025). AtBBX24 in *Arabidopsis thaliana*, GbBBX25 in *Ginkgo biloba* (heterocytically expressed in poplar), and MdBBX10 in apple (overexpressed in *Arabidopsis thaliana*) have all been confirmed to enhance the tolerance of plants to salt stress, involving soluble sugar accumulation, enhanced antioxidant capacity, and up-regulation of stress-related genes (Indorf et al., 2007; Liu et al., 2019; Huang et al., 2021). Moreover, CmBBX24,CmBBX22 and CmBBX19 in *chrysanthemum*, BBX18 in tomato, and MdBBX7 in apple (*Malus domestica*), participates in drought resistance regulation, Among them, CmBBX22 and CmBBX19 act as negative regulatory factors, This demonstrates that the BBX gene can exert both positive regulatory effects and serve as a negative regulator in plant drought responses (Yang et al., 2014; Xu et al., 2020; Chen et al., 2022; Liu et al., 2022; Li et al., 2024).

**Table 1:** Representative BBX involved in abiotic stress response.

Stress type	Species	Protein	Mechanism/target
Salt	*Triticum aestivum* L.	TaDBB4.1	reduced malondialdehyde (MDA) content
		TaDBB4.2	reduced malondialdehyde (MDA) content
	*Solanum lycopersicum* L.	SlBBX20	sustaining leaf photosynthetic capacity, reducing ROS accumulation, and elevating antioxidant enzyme activity and proline content
	*Arabidopsis thaliana*	AtBBX24	enhanced salt tolerance
	*Ginkgo biloba* L.	GbBBX25	Enhance soluble sugar and POD activity, and upregulate stress-related genes
	M. × domestica	MdBBX10	Enhance salt tolerance
drought	*Chrysanthemum morifolium* cv Fall Color	CmBBX24	Enhance drought resistance
	*Chrysanthemum morifolium*	CmBBX22	Negative regulation enhance drought resistance
	*Chrysanthemum morifolium*	CmBBX19	Negative regulation enhances drought tolerance
	*Solanum lycopersicum* L.	SlBBX18	Enhance drought resistance
	*Malus domestica*	MdBBX7	Enhance drought resistance
	*M. × domestica*	MdBBX10	Enhance drought tolerance
Cold	*Chrysanthemum morifolium* cv Fall Color	CmBBX24	Enhance cold resistance
	*Nicotiana tabacum* L.	NtBBX9	Inhibition of cold-responsive genes such as CBFs and increased ROS
		NtBBX11	Activating cold-responsive genes such as CBFs to reduce ROS
	*Arabidopsis thaliana*	AtBBX7/8	Activated by HY5, it regulates cold-responsive genes
	*Prunus persica* L. Batsch	PpBBX3,6,12,15,20,26	The expression level was positively correlated with the cold resistance of fruit
	*Malus domestica*	MdBBX37	Promote the expression of cold-responsive genes MdICE1 and MdCBF1
High temperature	*Solanum lycopersicum* L.	SlBBX17	Up-regulate SlHSF/SlHSP to enhance antioxidant defense
	*Lilium brownii* var. viridulum Baker	LlBBX15	Enhanced heat resistance
	*Vitis vinifera* L.	VvBBX32	It interacts with VvBBX8 and VvBBX11 to activate VvBZR1 and promote anthocyanin synthesis

Low-temperature stress seriously affects the geographical distribution of plants and the quality of crops. The BBX gene regulates cold tolerance by regulating core cold response pathways such as CBF and the balance of reactive oxygen species (ROS). In tobacco, NtBBX11 positively regulates cold resistance, while its homologous gene NtBBX9 acts as a negative regulatory factor. Both finely regulate the cold response by differentially regulating the expression of genes such as CBFs, LEA14, and LTI65, as well as the generation of ROS (Liu et al., 2023). Arabidopsis AtBBX7 and AtBBX8 are activated by HY5 and can positively regulate frost resistance (Li et al., 2021). Post-harvest low-temperature storage studies of peach fruits have shown that the expressions of PpBBX3, 6, 12, 15, 20, and 26 are significantly negatively correlated with the occurrence of cold damage, suggesting that they are involved in the low-temperature adaptation of fruits (Fang et al., 2021). Apple MdBBX37 enhances cold resistance by promoting the expression of MdICE1 and MdCBF1 (An et al., 2021).

High-temperature stress leads to protein denaturation and ROS outbreak. The BBX gene alleviates heat damage by activating the expression of heat shock proteins (HSP) and enhancing antioxidant defense. Tomato SlBBX17 was identified as a positive regulatory factor for heat tolerance. Overexpressed strains enhance heat tolerance by maintaining membrane stability, increasing antioxidant enzyme activity, reducing ROS accumulation, and up-regulating the expression of SlHsf and SlHSP genes (Xu et al., 2022a). In Lilium longiflorum, researchers identified a heat-inducible BBX gene, LlBBX15. Stable overexpression of LlBBX15 in Arabidopsis and Lilium significantly enhanced plant heat tolerance, whereas silencing this gene in Lilium resulted in reduced heat tolerance (LlBBX15, as a positive regulation, works in synergy with LlbHLH87 to act on the heat shock transcription factor signaling pathway, jointly promoting the establishment of plant heat tolerance) (Wu et al., 2026). In grapes, VvBBX32 and its interacting proteins VvBBX8 and VvBBX11 were found to jointly promote anthocyanin accumulation by activating the expression of the key factor *VvBZR1* in the brinolide signaling pathway under heat stress (Qiu et al., 2025), in order to counteract the inhibition of fruit coloring by high temperature. .

BBX proteins act as central integrators of developmental and environmental signals. Under abbiotic stresses such as salinity, drought, and extreme temperatures, BBX transcription factors regulate metabolic adaptation by regulating the accumulation of osmotic substances (such as proline and soluble sugars), maintain photosynthetic capacity, and enhance antioxidant defense by increasing the activity of enzymes such as SOD, POD, and CAT to reduce ROS damage. Meanwhile, they perform transcriptional control over the core stress signal cascade, including CBF-dependent cold response pathways, heat-resistant HSF, HSP modules, and hormone signaling pathways such as brassinolide signaling. Through these mechanisms, BBX proteins maintain cellular redox balance, preserve membrane integrity, and activate resistance genes to fine-tune plants adaptive responses to specific stressor effects.

### 6.2 Role of BBX proteins in biological stress response

**Figure 6:**
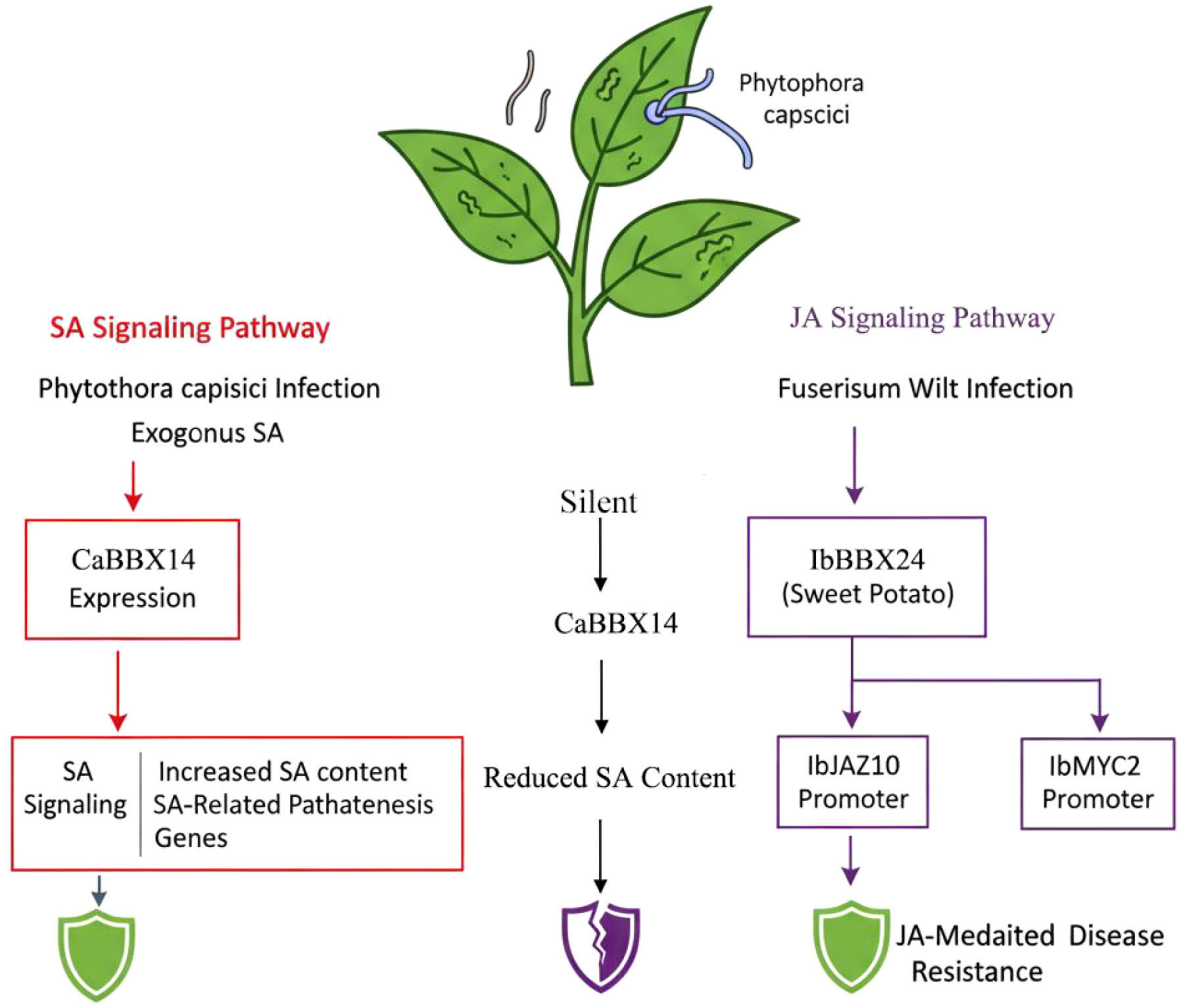
BBX-mediated plant disease resistance network. Purple indicates the JA pathway, red represents the SA pathway, black denotes the silencing pathway, and arrows denote activation effects.A fully green shield represents resistance to diseases, while a cracked purple shield indicates disease invasion.

In addition to abiotic stress, BBX proteins are also involved in the defense responses of plants against biological stress such as pathogen infection. Its function is often intertwined with hormone signaling pathways, especially the salicylic acid (SA) and jasmonic acid (JA) pathways (**Figure 6**). In *Capsicum annuum* L. ‘Hangjiao12’, the expression of CaBBX14 was significantly induced by infection with Phytophthora capsici and exogenous salicylic acid (SA) treatment (Zhou et al., 2023). Functional studies have shown that silencing CaBBX14 reduces the plant’s resistance to Phytophthora, accompanied by a decrease in SA content and the expression of SA-related pathogenic genes, indicating that CaBBX14 positively regulates disease resistance mediated by the SA signaling pathway. Similarly, sweet potato IbBBX24 has been demonstrated to be involved in JA-mediated resistance to Fusarium wilt by binding to the promoters of IbJAZ10 and IbMYC2 (Zhang et al., 2020). Furthermore, multiple *BvBBX* genes of sugar beet (*Beta vulgaris* L.) display differential expression patterns during Cercospora leaf spot infection (Song et al., 2023). These findings reveal the regulatory role of BBX transcription factors in the plant immune system.

## 7 Conclusions and perspectives

In conclusion, BBX transcription factors, as multifunctional regulatory hubs, precisely regulate downstream gene networks by responding to light, hormone and stress signals, and coordinate plant growth and development with adaptation to adverse conditions.

Previous studies have demonstrated the critical importance of BBX proteins in plants. By employing modern breeding techniques, including gene editing and molecular marker-assisted selection, researchers have developed new crop germplasms characterized by ideal plant morphology, superior fruit coloration, high nutritional quality, and enhanced resistance to multiple stresses, such as salt-alkali tolerance, drought, cold, heat, and diseases. Although the essential role of the BBX transcription factor family in integrating light and stress signals, as well as regulating plant growth and development, has been initially elucidated, and functional genes with significant breeding potential have been identified across various crops, several key scientific bottlenecks remain in effectively translating this foundational knowledge into precision breeding tools. First, a systematic analysis of the functional redundancy of the BBX protein and its interaction network is essential to elucidate the relative relationships among BBX proteins. Currently, our understanding of the functional overlap and differentiation mechanisms among BBX family members remains incomplete, particularly regarding the composition of protein complexes, upstream regulatory factors, and downstream signal transduction networks. These areas necessitate further exploration and validation through multidimensional experimental approaches. To achieve this, the biological functions of the BBX protein can be systematically characterized by constructing collections of single mutants and higher-order multi-mutants. Building on this foundation, additional research should focus on the functions of the BBX protein across various crops. It is important to establish classifications for BBX proteins, including conserved types (highly conserved sequences and functions), species-specific types (unique to particular species), and variant types (notable sequence variations), and to develop precise editing site libraries for these three categories. Furthermore, high-throughput technologies such as DAP-seq and ChIP-seq should be employed to identify the target genes directly regulated by BBX proteins, thereby facilitating the establishment of a refined transcriptional regulatory network. Proteomics techniques, including Co-IP-MS and IP-MS, were employed to identify and validate the protein complexes that interact with BBX, resulting in the construction of a comprehensive protein interaction system. This research culminated in the development of an extensive regulatory and interaction network for BBX, encompassing multiple levels of ‘DNA-protein-protein’ interactions. This network establishes a foundational platform for the targeted manipulation of BBX functions in precision breeding. Furthermore, it is essential to elucidate the integrated response mechanisms within complex field environments and to demonstrate the efficient and precise regulatory capabilities of BBX proteins. Most existing studies have concentrated on simulating controlled environments under single stress conditions, which inadequately represent the multifactorial synergistic or antagonistic effects encountered in natural settings. The molecular integration mechanisms of BBX proteins in response to the cross-adaptation of various biological and abiotic stresses, as well as their functional performance in actual agricultural ecosystems, require systematic investigation through multi-stress joint experiments and field validations. To address this, a multi-factor combined stress platform should be established to replicate the ecological conditions of real farmland, enabling the collection of ecological data (temperature, humidity, light intensity, CO_2_ concentration), soil data, and plant phenotypic data. Additionally, omics approaches should be utilized to elucidate the dynamic changes of BBX proteins in plants. By integrating and analyzing machine learning algorithms, including random forests and neural networks, this study investigates the key environmental factors that influence changes in BBX expression. A BBX-mediated environmental signal integration network model is constructed to facilitate this analysis. Additionally, the basic platform is enhanced under multiple coercive conditions. Through modeling, the dynamic output of the network is simulated in response to various stresses, specifically under conditions of gene deletion or overexpression. This approach enables the prediction of optimal genes and the best editing targets for the cultivation of ideal plant types. Furthermore, network computing simulation analysis identifies core BBX proteins at critical intersection points, such as stress resistance versus yield, plant height versus yield, yield versus quality, and space versus yield. By employing gene editing technology, it is possible to balance key agronomic traits, including yield and quality, while simultaneously enhancing stress resistance. In conclusion, the establishment of a comprehensive precise breeding application system for BBX proteins can significantly advance the goal of breeding horticultural crops.

## Data Availability

The original contributions presented in the study are included in the article/supplementary material. Further inquiries can be directed to the corresponding authors.
